# Transcriptome responses to different herbivores reveal differences in defense strategies between populations of *Eruca sativa*

**DOI:** 10.1186/s12864-019-6217-9

**Published:** 2019-11-12

**Authors:** Ariel Ogran, Adi Faigenboim, Oz Barazani

**Affiliations:** Institute of Plant Science, Agricultural Research Organization – the Volcani Center, 7505101 Rishon LeZion, Israel

**Keywords:** RNA Seq, Jasmonic acid, Salicylic acid, Glucosinolates, Generalist vs. specialist insects

## Abstract

**Background:**

Intraspecific variations among induced responses might lead to understanding of adaptive variations in defense strategies against insects. We employed RNA-Seq transcriptome screening to elucidate the molecular basis for phenotypic differences between two populations of *Eruca sativa* (Brassicaceae), in defense against larvae of the generalist and specialist insects, *Spodoptera littoralis* and *Pieris brassicae*, respectively. The *E. sativa* populations originated from desert and Mediterranean sites, where the plants grow in distinct habitats.

**Results:**

Responses to elicitation of the plants’ defenses against wounding and insect herbivory resulted in more upregulated transcripts in plants of the Mediterranean population than in those of the desert. PCA analysis differentiated between the two populations and between the elicitation treatments. Comprehensive analysis indicated that defense responses involved induction of the salicylic acid and jasmonic acid pathways in plants of the desert and Mediterranean populations, respectively. In general, the defense response involved upregulation of the aliphatic glucosinolates pathway in plants of the Mediterranean population, whereas herbivory caused downregulation of this pathway in desert plants. Further quantitative RT-PCR analysis indicated that defense response in the desert plants involved higher expression of nitrile-specifier protein (*NSP*) than in the Mediterranean plants, suggesting that in the desert plants glucosinolates breakdown products are directed to simple-nitriles rather than to the more toxic isothiocyanates. In addition, the defense response in plants of the desert population involved upregulation of flavonoid synthesis and sclerophylly.

**Conclusions:**

The results indicated that differing defense responses in plants of the two populations are governed by different signaling cascades. We suggest that adaptive ecotypic differentiation in defense strategies could result from generalist and specialist herbivore pressures in the Mediterranean and desert populations, respectively. Moreover, the defense responses in plants of the desert habitat, which include upregulation of mechanical defenses, also could be associated with their dual role in defense against both biotic and abiotic stresses.

## Background

The term “induced defense” in plants refers to their ability to respond to herbivory by elevating their defense mechanisms, which rely mainly on the jasmonic acid signaling cascade and its interactions with other phytohormones, mainly salicylic acid and ethylene [1, 2]. Consequently, to optimize their defenses plants differ in their responses to different herbivores or to wounding in general, e.g., [3–7]. Such differential responses need the plant to recognise specific attacks, via mechanisms that mainly are associated with specific elicitors in the oral secretions of the chewing insect [8–10].

In members of the Brassicaceae, induced resistance against herbivory includes accumulation of glucosinolates, the main chemical defense metabolites, which provide effective defense against a wide range of herbivores [11, 12]. Mechanical damage to the leaf, caused by a herbivore, releases the enzyme myrosinase, which hydrolyzes intact glucosinolate molecules to their bioactive breakdown products: epithionitriles, nitriles, thiocyanate, and more-toxic isothiocyanates (ITC). However, specialist herbivores such as *Pieris rapae* and *Plutella xylostella* evolved mechanisms to suppress the release of the toxic products of glucosinolates breakdown and thereby to avoid the harmful effects of ITC [13–15]. Moreover, in *Arabidopsis*, it was shown that herbivory by the specialist *P. rapae* did not induce accumulation of aliphatic glucosinolates, as herbivory by the generalist *Spodoptera exigua* did [16]. In support of this specialist/generalist paradigm, it also was shown that specialist and generalist chewing herbivores elicited different phytohormone responses, as in the case of *Boechera divaricarpa* (Brassicaceae) [7]. However, contrary to this generalist/specialist paradigm, a microarray analysis of *A. thaliana* showed that plant responses to various specialist and generalist lepidopteran species could not be attributed to the insects’ degree of specialization [17].

It is assumed that the level of herbivory can lead to spatial intraspecific variation in defense against herbivores; more strongly defended plants are common in habitats where herbivores are more dominant, and vice versa [18]. In consistent with this general concept, chemotypic variation in glucosinolates profiles among 75 populations of *A. thaliana* in Europe was strongly correlated with the abundance of the specialist aphids *Brevicoryne brassicae* and *Lipaphis erysimi* [19]. Other studies have shown that genetic variations in defenses were associated with plants’ competitive ability [20, 21], which suggests that response to herbivores may also mediate allelopathic interactions [21, 22]. These studies strengthen the general view of the optimality theory [23] in that they stress the role of the tradeoff between growth and defense in shaping genetic variation of induced compared with constitutive defenses [20]. In addition, because herbivores are expected to prosper in competitive plant communities characterized by high vegetation cover, variation in herbivore communities may represent a selective pressure that leads to intraspecific variations of defenses [19].

Previously we showed that in Israel, populations of *Eruca sativa* (Brassicaceae) originating from Mediterranean and desert habitats, differed in their induced defenses against specialist and generalist herbivores, i.e., *P. brassicae* and *S. littoralis*, respectively [5]. Herbivory by larvae of the generalist and the specialist insects induced accumulation of glucosinolates in plants of the Mediterranean population but not in those of the desert population. Furthermore, our previous results suggested that trypsin proteinase inhibitor activity was involved in the induced defense response in plants of the desert population [5]. To further test our hypothesis that populations of *E. sativa* exhibit ecotypic differentiation in defense strategies against herbivores, in the present study we applied RNA-Seq technology to analyse molecular patterns associated with herbivory in plants of the two populations. Most previous studies used transcriptome screening to examine the molecular basis of the responses of cruciferous species to various herbivores [3, 5–7]. In the present study a molecular comparison between plants of the two populations provided a more comprehensive understanding of the global transcript patterns and specific genes relevant to responses of plants to specific treatments (i.e., exposure to larvae of the generalist *S. littoralis* and specialist *P. brassicae*). It also enabled comparison between the effects of different environments which, in turn, can be linked to counter-adaptation of plant populations to different herbivores.

## Methods

### Plant growth conditions

Seeds of two populations, characteristic of desert (32° 04′ 49“ N, 35° 29’ 46” E; ≤ 200 mm annual rainfall) and Mediterranean (32° 46′ 39“ N, 35° 39’ 29” E; ≥430 mm annual rainfall) habitats, created under uniform conditions [24], were germinated on moistened Whatman No. 1 filter paper in 9-cm Petri dishes. The seeds were set to germinate in a growth chamber at 25 °C with an 8/16-h day/night photoperiod. Four-day-old seedlings were transferred to germination trays and placed in an insect-free net house; after 2 weeks the plants were transferred to 1-L pots containing a mixture of 50% peat, 30% tuff, and 20% perlite (Shacham, Israel) and were irrigated with an automatic irrigation system at 150 mL/day per pot. The experiment was conducted in February and March, with average max/min temperatures of 20/14 °C.

### Elicitation and sampling

Defense mechanisms in the plants of the two investigated populations were induced by: 1) wounding with a pattern wheel; 2) wounding and application of 20 μL of oral secretions (OS) of *S. littoralis* and *P. brassicae*, diluted 1:5 (v:v) with distilled water. In the first group of plants, distilled water was applied to the wounded leaves (designated as “wounded”). Fivefold-diluted OS of *S. littoralis* was shown to cause transcript change in plants of *A. thaliana* [3]. Similarly, preliminary experiments (not shown) and further quantitative RT-PCR tests [see Results section] showed that the diluted OS of both the generalist and the specialist larvae caused transcriptional changes as compared with control and wounded leaves. Treatment with OS enabled collection of leaf samples up to 48 h after elicitation without reduction of tissue area, as found when larvae were reared directly on the plants. To collect the OS, larvae of the generalist (*S. littoralis*) and specialist (*P. brassicae*) herbivores were reared from hatching to the third-instar stage on *E. sativa* plants, and their OS were collected with a vacuum system.

Five leaves on each plant were elicited, and were harvested at several time points after elicitation, from 0 (control) to 48 h. They were collected in chronological order starting at the first elicitation time point, and at each time point one leaf was harvested from every plant, all at the same development stage; an additional leaf was collected from each non-induced plant, as a control. The samples were immediately frozen in liquid nitrogen and were further used for RNA isolation.

### RNA isolation and RNA-Seq analysis

Total RNA of each collected sample was extracted by using the TRI reagent (Sigma-Aldrich, Israel); 1 μL (2 u) of DNAse (Ambion, Thermo Fisher Scientific) was added to each sample to remove traces of DNA. RNA quality and integrity were verified with the Tapestation 2200 system (Agilent Technologies, USA). A 3-μg sample of RNA from each of the three leaves of a single plant was pooled to create one sample of the early (i.e., taken after 0.5–2 h) elicitation response (ER) to each treatment. Late elicitation responses (LR) for each plant included a pooled samples of RNA extracted from leaves collected 24 and 48 h after elicitation (4.5-μg for each time point). Samples from each population comprised a total of six biological replicates per treatment — three for each of the early- and late-induced responses; a single plant was used as a biological replicate. The control samples comprised three replicates. Thus, there was a total of 42 analysed samples. cDNA libraries were then prepared with the TruSeq Library Prep Kit V (Ilumina, Inc.). The samples were sequenced with the Illumina Hi-Seq 2000 system at the Technion Genome Center (Haifa, Israel).

### De novo transcriptome assembly

Raw reads were subjected to a filtering and cleaning procedure as follow: First, the SortMeRNA tool was used to filter out rRNA [25]; then the FASTX Toolkit (http://hannonlab.cshl.edu/fastx_toolkit/index.html, version 0.0.13.2) was used for: (i) trimming read-end nucleotides with quality scores < 30 by using the fastq_quality_trimmer; (ii) removing reads with less than 70% of base pairs with quality score ≤ 30 by using the fastq quality filter. The total of ~ 1 T cleaned reads, obtained after processing and cleaning, were assembled de novo by using the Trinity software (version trinityrnaseq_r20140717 2.1.1) [26]. The resulting de novo-assembly-generated transcriptome consisted of 80,946 contigs with N50 of 1081 bp.

### Data availability

The sequencing data were deposited in the NCBI Sequence Read Archive (SRA) database as bioproject PRJNA511735.

### Sequence similarity and functional annotation

To assess the similarity of the transcriptome to those of other models and closely related species, sequence similarity was analyzed with the BLAST (Basic Local Alignment Search Tool) algorithm with an E-value cut-off of 10^− 5^ [27]. The BLASTX algorithm was used to search protein databases by using a translated nucleotide query for comparison of the assembled contigs with sequences deposited in the *Arabidopsis* information resources (TAIR, http://www.arabidopsis.org). The transcriptome was used as a query for a search of the NCBI non-redundant (nr) protein database that was carried out with the DIAMOND program [28].

### Differential expression and cluster analysis

The transcript was quantified (i.e., the number of reads per gene was determined) from RNA-Seq data by the using Bowtie aligner [29] and the Expectation-Maximization method (RSEM) [30]. Differential expression was analysed with the edgeR software suit [31] and transcripts that were more than twofold differentially expressed with false-discovery-corrected statistical significance of ≤0.05 were considered differentially expressed [32]. The expression patterns of the transcripts in different samples were studied by using cluster analysis of the differentially expressed transcripts in at least one pairwise sample comparison. Then, the Trinity protocol [33] was used to design expression normalization by using TMM (trimmed mean of M-values), following calculation of FPKM (fragments per feature kilobase per million). Hierarchical clustering of the normalized gene expression (by using centralized and log2 transformation [33]) and heat-map visualization were performed by using R Bioconductor [34]. The VENNY tool [35] was used for construction of Venn diagrams. Principal component analysis (PCA) was applied with the FactoMineR package of R [36] to gain more insight on separation between samples of each treatment separately (based on the normalized expression of the average of three replications).

### GO enrichment and pathways analysis

Gene ontology (GO) enrichment analysis was carried out by using the Plant MetGenMAP (http://bioinfo.bti.cornell.edu/cgi-bin/MetGenMAP/home.cgi) [37], based on the TAIR homology results. The tool enables integration of the functional categories between populations and among treatments with multiple testing correction of False Discovery Rate (FDR < 0.05) [32]. Differentially expressed genes were displayed on diagrams of the Secondary Metabolism Map by using MapMan [38].

### Quantitative PCR

Quantitative RT-PCR was applied for further analysis of two myrosinase-associated proteins — the nitrile-specifier protein *NSP2* and the epithiospecifier modifier *ESM1* — as marker genes for glucosinolate breakdown [5]. Reverse transcription with oligo dT (Fermentas Thermo) was used to synthesize cDNA from RNA samples (see RNA isolation and RNA-Seq analysis above). The cDNA samples were diluted to a uniform concentration (62 ng/μL) and qRT-PCR amplifications were performed with a Rotor-Gene 6000 instrument (Corbett-Qiagen, Valencia, CA, USA) by using components supplied in the KAPA SYBR FAST kit (Kapa Biosystems, Woburn, MA, USA), as previously described [5]. The threshold cycle (Ct) was automatically determined with Rotor-Gene 6000 software and the relative expression levels of target genes were calculated with the aid of a ‘two-standard curve’ (i.e., that of the gene of interest and that of actin), implemented in the Rotor-Gene software. Each sample was analysed in two technical replicates for each target gene. Standard curves were created in each run by using a pooled cDNA sample; a reference cDNA calibration sample was used to normalize the multi-run results.

## Results

Quality trimming and filtration resulted in a total of ~ 1 T cleaned reads with an average of 22.3 M clean reads per sample. These were assembled by using Trinity, and generated 80,946 contigs for the transcriptome catalogue, with N50 of 1081 bp. Matching against the TAIR database yielded a significant hit of 54,552 contigs (67.4%). Annotating the transcriptome catalogue by aligning the contigs to the NCBI non-redundant (nr) protein database resulted in 61,403 out of 80,946 contigs (75.85%), with at least one DIAMOND hit to a protein. Of these contigs, 30,251 (~ 50%) matched sequences from the genomes of *Brassica napus*, followed by ~ 20% matching with *Brassica oleracea var. oleracea* (Additional file 1: Figure S1). A summary of the transcriptome catalogue, presenting the information of the full assembly and the number of raw reads, clean and mapped reads of each sample is provided in Additional file 4.

**Fig. 1:**
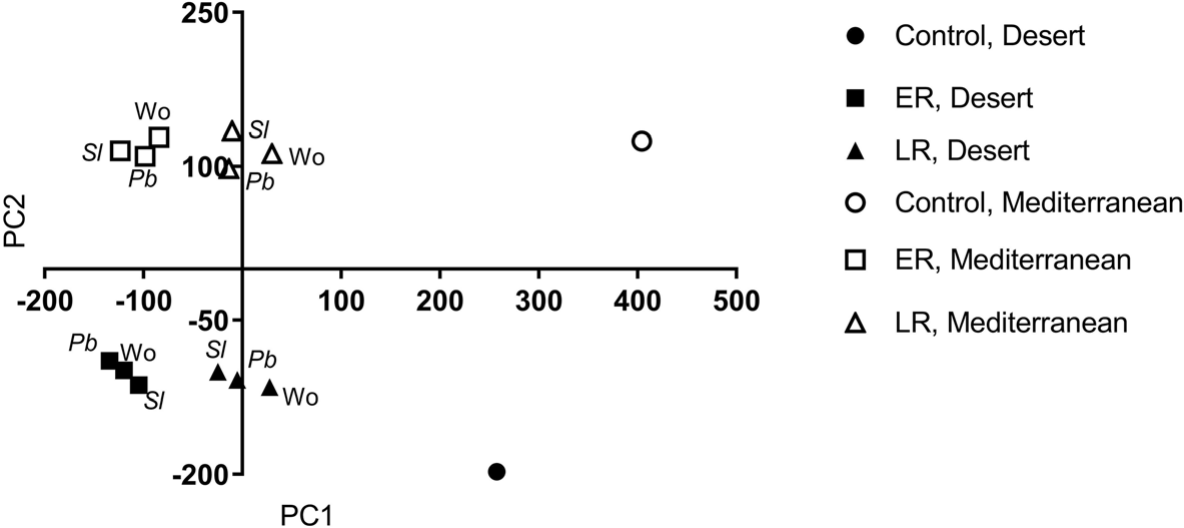
Results of the principal component analysis (PCA) of the transcriptome profiles of *E. sativa* populations. Control, early (ER) and late (LR) responses to the three elicitation treatments: wounding (Wo) or OS of *S. littoralis* (*Sl*) or *P. brassicae* (*Pb*). The first two axes account for 27.15% (PC1) and 17.86% (PC2) of the variation

**Fig. 2:**
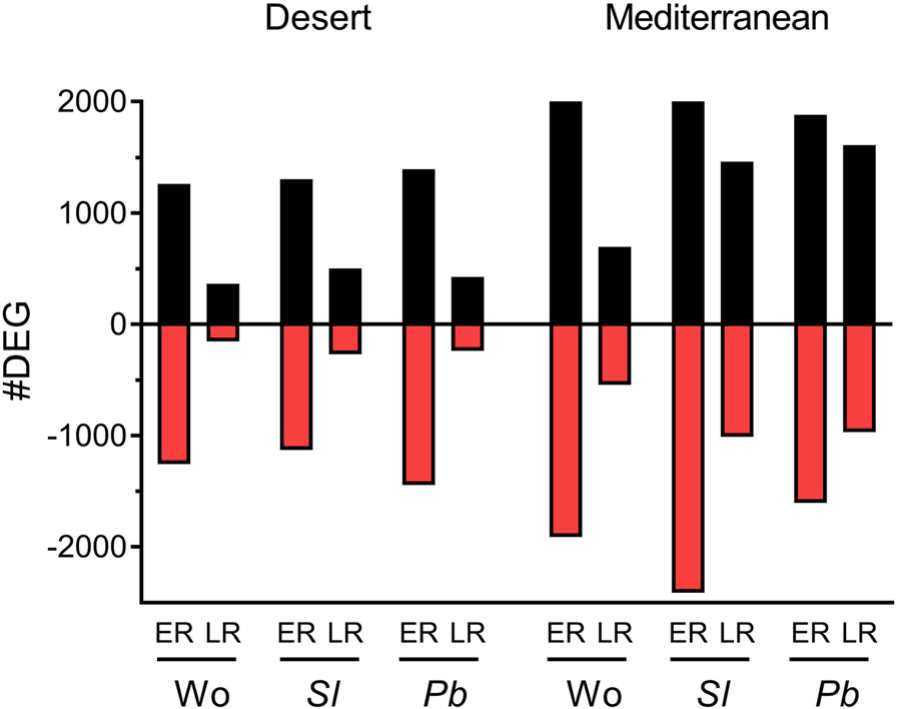
Numbers of differentially up- and downregulated genes. Numbers of differentially upregulated (black) and downregulated (red) genes (DEG) in plants of the desert and Mediterranean populations, as compared with control non-elicited plants. Results present the early and late responses (ER and LR, respectively) to wounding (Wo) or to OS of *S. littoralis* (*Sl*) or *P. brassicae* (*Pb*)

Principal component analysis (PCA) divided the transcriptome profiles mainly according to population and time after elicitation (Fig. 1 and Additional file 2: Figure S2). The first two axes of the PCA represent the differentiation between the two populations and between the controls and ER and LR of the three elicitation treatments (Fig. 1). Analysis of the number of differentially expressed genes (DEGs) derived for each elicitation treatment were determined according to their significance (*FDR* < 0.05; twofold) as compared with control non-elicited plants. Overall, the results revealed that the ER to wounding or to herbivory by *S. littoralis* and *P. brassicae* yielded more transcriptional changes than the LR (Fig. 2). Elicitation by wounding, *S. littoralis* and *P. brassicae* caused substantially more changes in the numbers of ER-upregulated DEGs in the Mediterranean plants than in those of the desert population — 1.7-, 1.8- and 1.3-fold, respectively.

### Comparison of the transcript patterns between treatments: co-expressed and treatment-specific DEGs

**Fig. 3:**
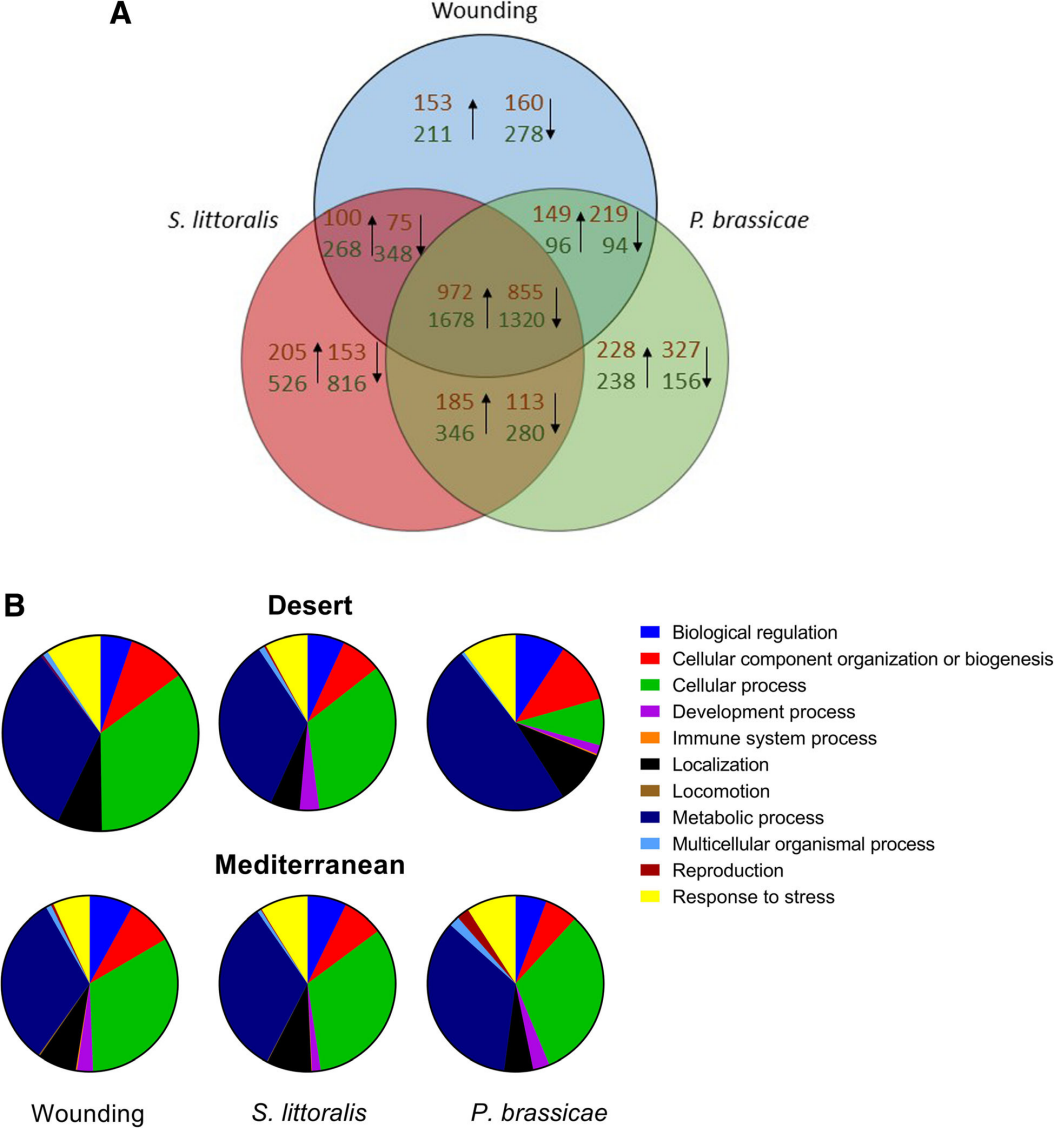
Venn diagrams and pie diagrams. **a** Venn diagrams, representing the numbers of overlapping and non-overlapping significantly up- and downregulated DEGs in plants of the desert (red fonts) and Mediterranean (green fonts) populations; **b** Pie diagrams representing the categorization of upregulated exclusive DEGs of the various elicitation treatments to different biological processes (based on MetGenMAP functional classification)

Venn diagrams enabled clustering of the DEGs into up- and downregulated genes of overlapping and treatment-specific groups, relative to unelicited control samples (Fig. 3a). Many DEGs were co-expressed by the three elicitation treatments in plants of the desert and Mediterranean populations, both up- and downregulated DEGs. The percentage of upregulated DEGs specifically elicited by OS of *S. littoralis* was higher in the Mediterranean plants than in the desert ones: 18.7 and 14.0%, respectively. The responses to OS of the generalist herbivore also resulted in a higher percentage of downregulated DEGs in the Mediterranean plants than in the desert ones, at 29.5 and 12.8%, respectively. But the response of plants to elicitation by the specialist herbivore yielded higher percentages of non-overlapping up- and downregulated DEGs in the desert plants — 14.9 and 21.6%, respectively — than in the Mediterranean ones — 10.1 and 8.4%, respectively (Fig. 3a).

To improve our understanding of the differences between the responses of plants of the two populations to the elicitation treatments, we then analyzed gene ontology (GO) to cluster the core set of treatment-specific upregulated and downregulated DEGs to biological processes. Overall, in plants of both populations most of the differentially upregulated DEGs in the three elicitation treatments were categorized to metabolic or cellular processes (Fig. 3b). Nevertheless, following exposure to OS of the generalist herbivore, slightly more treatment-specific upregulated genes were associated with defense responses (categorized as ‘response to stress’) in the Mediterranean plants than in the desert ones: 8.8 and 7.9%, respectively (Fig. 3b).

Classification according to pathways categorized the total non-overlapping DEGs of plants of the Mediterranean and desert populations to a maximum of 159 upregulated pathways in Mediterranean plants and 95 in desert plants, in response to OS of *S. littoralis* and *P. brassicae*, respectively. The numbers of downregulated non-overlapping pathways were highest in the desert and Mediterranean plants following treatment with OS of the specialist and generalist herbivores, respectively: 203 and 262 pathways, respectively. The complete list of up- and downregulated pathways is presented in Additional file 5.

**Fig. 4:**
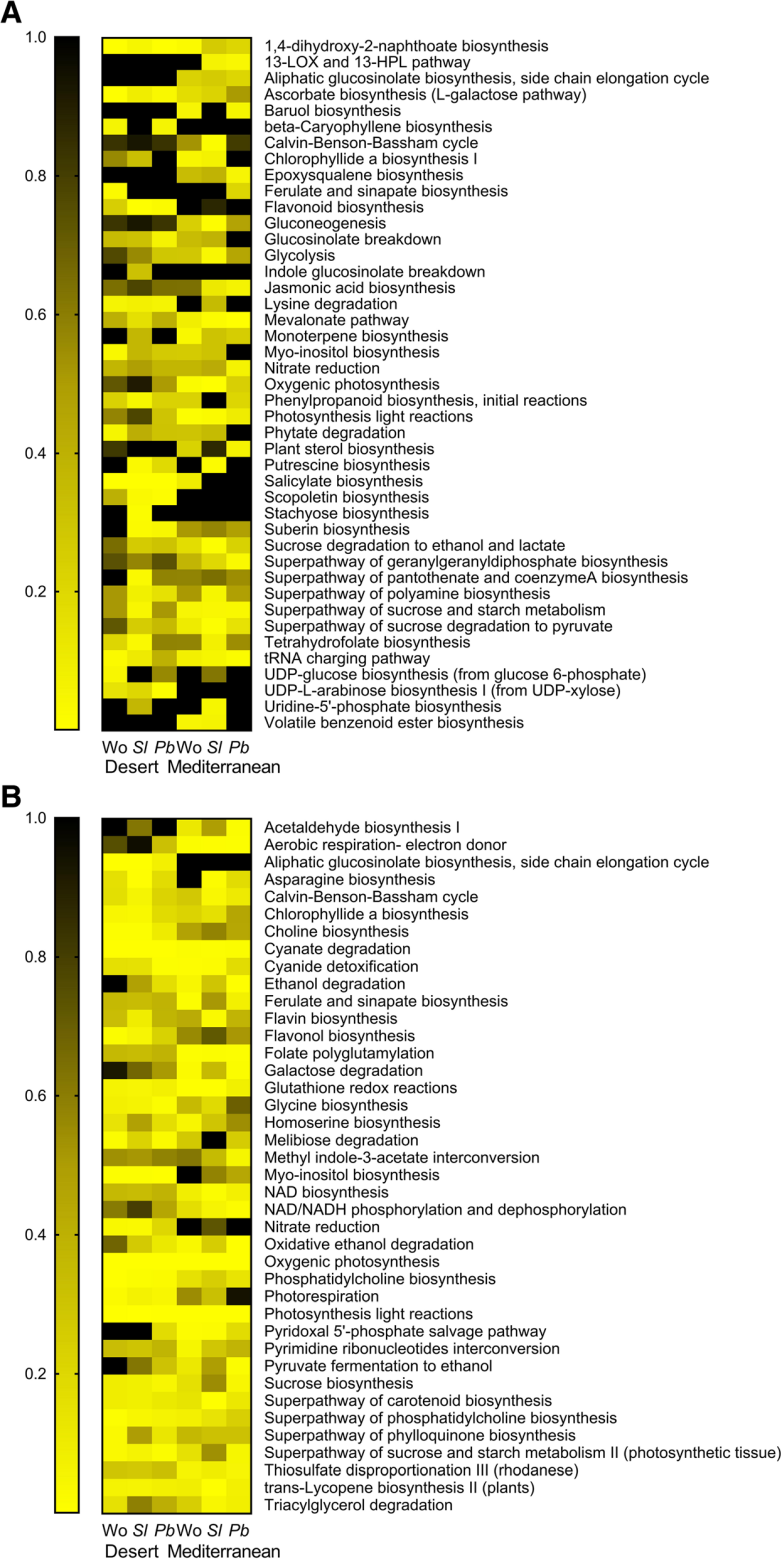
Heat-maps presenting the strength of *P* values. Heat-maps presenting the strength of *P* values of the significantly up- (**a**) and downregulated (**b**) pathways in the non-overlapping groups of the various elicitation treatments (cf. Figure 3)

Figure 4 presents a heat-map of the *P* values of pathways that were significantly changed in at least one treatment; it shows that salicylate and phenylpropanoid biosynthesis were among the pathways in plants of the desert population that were significantly changed by all elicitation treatments. In addition, treatment with OS of both the generalist and specialist herbivores significantly induced the flavonoid (*P* < 0.01) and suberin (*P* < 0.05) biosynthesis pathways in plants of the desert population (Fig. 4a). The results also indicated that glucosinolate breakdown was significantly changed in response to OS of the specialist herbivore only in the desert plants (*P* = 0.05). In Mediterranean plants the mevalonate pathway was significantly upregulated by all elicitation treatments, and the jasmonic acid pathway was significantly changed in response to treatment with OS of both the generalist and the specialist herbivores. Treatment with OS of the generalist insect significantly changed the putrescine biosynthesis pathway in plants of both populations (Fig. 4a).

Most of the downregulated pathways encompassed primary metabolic processes: photosynthesis and sucrose biosynthesis, among others (Fig. 4b). Interestingly, the aliphatic glucosinolate biosynthesis pathway was significantly downregulated in plants of the desert population, in response to wounding and to elicitation by OS of *S. littoralis* (Fig. 4b).

### Differences between populations of *E. sativa*, in their transcriptome profiles

**Fig. 5:**
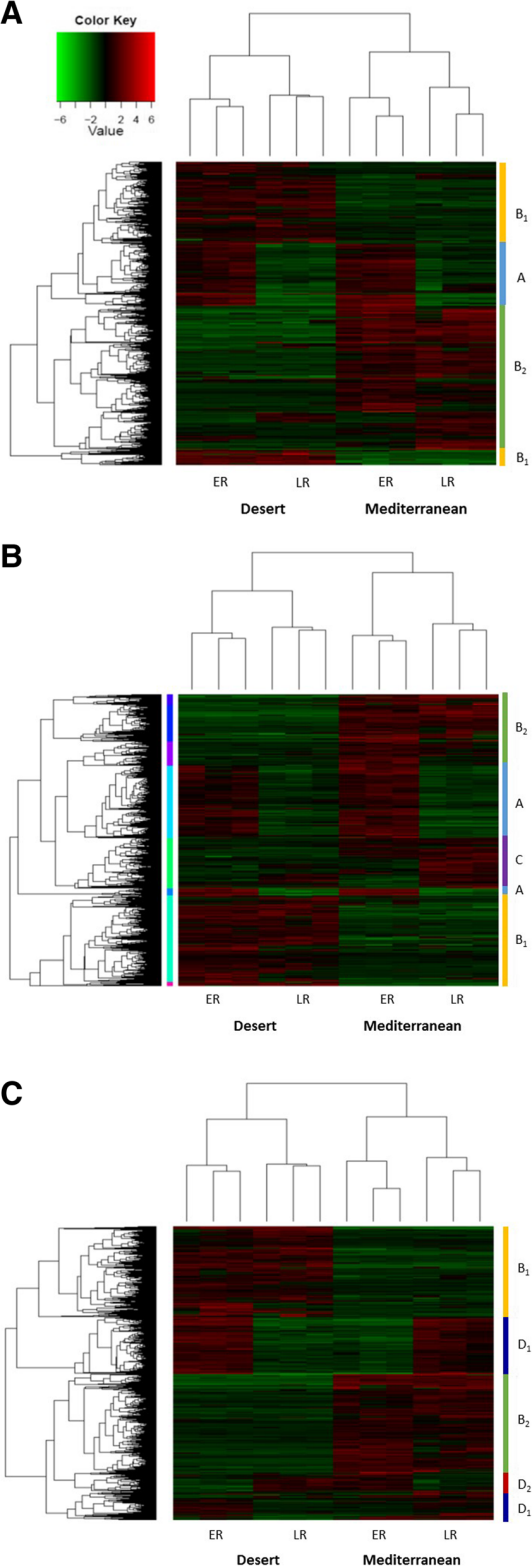
Heat-maps representing the clustering of transcript profiles. Heat-maps representing the hierarchical clustering of the significantly differential expressed transcripts of the early and late responses (ER and LR, respectively) of the three replicates of the two populations, following wounding (**a**), or OS of *S. littoralis* (**b**) and *P. brassicae* (**c**)

Clustering in heat-maps of the significantly differentially expressed transcripts, which differentiated between the transcriptome profiles of the two populations and between ER and LR (Fig. 5), aided interpretation of the differences between the responses of plants of the two populations to the elicitation treatments. In general, the transcriptome profiles could be divided into two main groups of gene clusters: Transcripts in the first group differentiated similarly between early and late responses in plants of both populations (clade designated as ‘A’, Fig. 5a, b). The second group included transcripts that differentiated between the transcriptome profiles of plants of the two populations (i.e., clade B in Fig. 5a and b, clade C in Fig. 5b and clade D in Fig. 5c). In the second group: Clade B_1_ clustered transcripts that were upregulated in the desert plants but downregulated in the Mediterranean ones and vice versa for B_2_ (Fig. 5a, b); Clade C mostly clustered LR transcripts that were exclusively upregulated in response to elicitation by OS of *S. littoralis* in Mediterranean plants (Fig. 5b); Clade D differentiated between the transcriptome profiles of the ER and the LR of plants of both populations that were elicited with OS of the specialist herbivore (Fig. 5c).

**Fig. 6:**
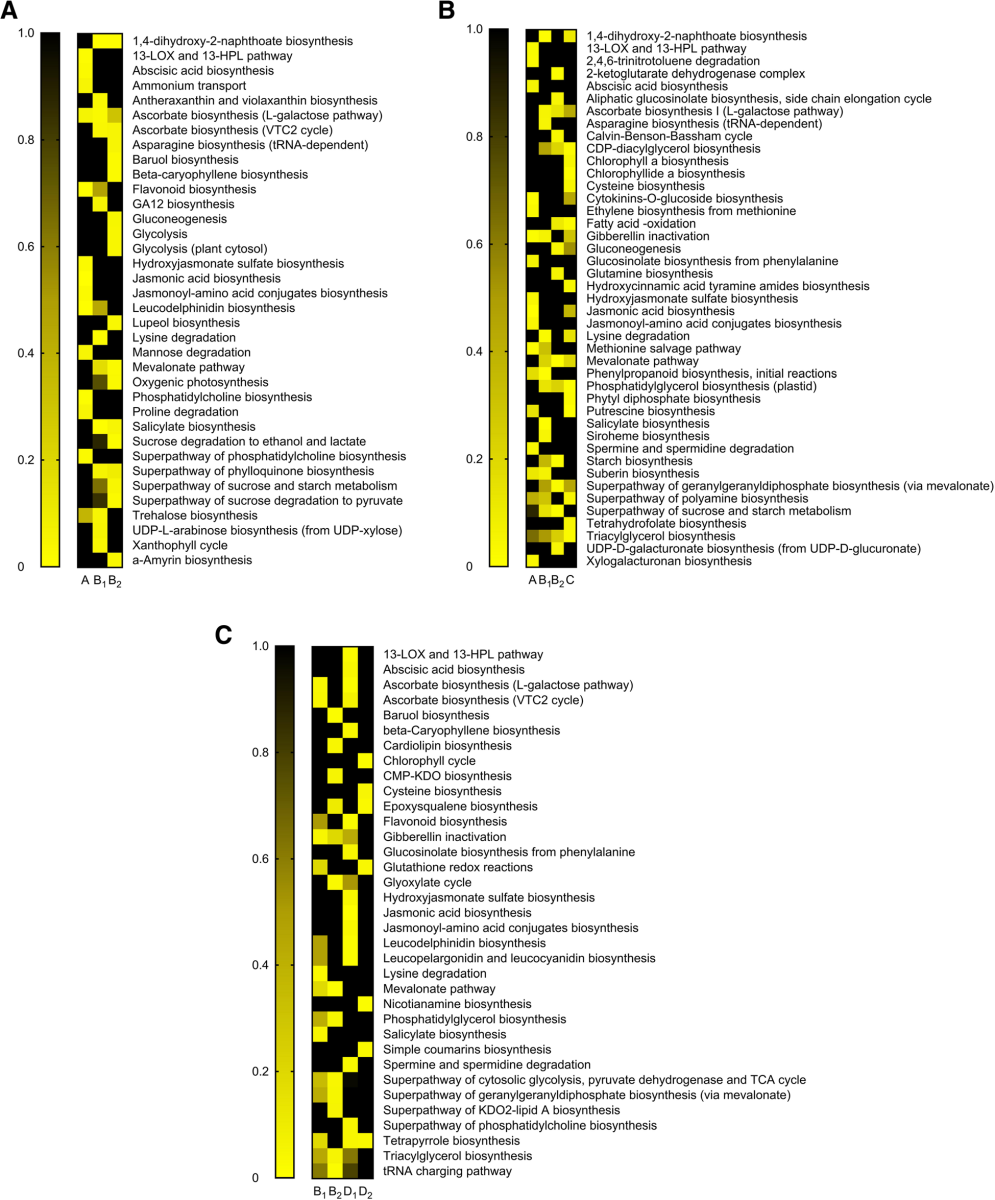
Categorization of transcripts of the various clades. Categorization of transcripts of the various clades (cf. Figure 5) of leaves elicited by wounding (**a**), or OS of *S. littoralis* (**b**) and *P. brassicae* (**c**) to pathways, based on strength of the *P* value (heat-maps)

Clade A, which differentiated between the upregulated ER and the downregulated LR, in plants of both populations, included 359 and 658 transcripts from leaves that were either wounded or treated with OS of the generalist herbivore, respectively — 24.1 and 32.1%, respectively, of the total (Fig. 5a, b; Additional file 6). Following wounding, more transcripts were clustered in clade B_2_ than in B_1_ (538 and 447, respectively) (Fig. 5a). Further classification according to pathways showed that following wounding, the jasmonic acid pathway (*P* < 0.001) and its conjugates (*P* = 0.024), the LOX-HPL cascade (*P* = 0.01) and the abscisic acid (*P* = 0.023) and flavonoid biosynthesis (*P* = 0.001) pathways were among the significantly changed pathways in clade A (Fig. 6a; Additional file 6). The salicylate biosynthesis and the mevalonate pathways were among the significantly changed pathways in both clades B_1_ and B_2_ (Fig. 6a).

The transcriptome profile of plants that were treated with OS of the generalist herbivore (Fig. 5b) included 1.6 times as many transcripts in clade B_1_ as in B_2_ — 539 and 337 genes, respectively — whereas clade C clustered 426 LR genes that were mostly upregulated in Mediterranean plants — 20.7% of the total processed hits (Additional file 6). Glucosinolate biosynthesis from phenylalanine (*P* = 0.05) was among the significantly changed pathways of clade A, together with several signaling pathways such as jasmonic acid (*P* < 0.001) and its conjugates (*P* = 0.037), the LOX-HPL pathway (*P* = 0.025), and the ethylene (*P* = 0.023), abscisic acid (*P* = 0.05) and cytokinins biosynthesis (*P* = 0.002) pathways. The salicylate (*P* < 0.001), suberin (*P* = 0.05) and phenylpropanoid synthesis (*P* = 0.005) pathways were among the significantly changed pathways of clade B_1_; the aliphatic glucosinolates (*P* = 0.04) and the mevalonate pathways (*P* = 0.004) were among the significantly changed pathways of clade B_2_ (Fig. 6b; and Additional file 6).

Similar numbers of transcripts of the transcriptome profile of plants that were treated with OS of *P. brassicae* were clustered in clades B_1_ and B_2_; they comprised 31.3 and 28.6%, respectively, of the total processed hits (Fig. 5c; Additional file 6). However, 3.7 times as many transcripts were clustered in clade D_1_ as in D_2_; this differentiated the ER from the LR transcriptome of plants of the two populations. These were classified into glucosinolate biosynthesis from phenyalanine (*P* = 0.04), flavonoid synthesis (*P* = 0.02), and the signaling cascades: jasmonic acid synthesis (*P* < 0.001) and its conjugates (*P* = 0.034), and the LOX-HPL (*P* = 0.021) and abscisic acid synthesis (*P* = 0.042) pathways. The salicylate biosynthesis (*P* = 0.04) and the mevalonate pathways (*P* = 0.007) were among the significantly changed pathways in clades B_1_ and B_2_, respectively (Fig. 6c; and Additional file 6).

A MapMan representation of the comparison between expression patterns of genes in plants of the two population (Additional file 3: Figure S3) illustrates the differences between responses to the three elicitation treatments, as well as differences between the responses of plants to the elicitation treatment. The results highlighted the defense roles of glucosinolates and terpenoids in Mediterranean plants, and that of flavonoids in desert plants. The results showed that OS of the specialist herbivore activated differing response in plants of the two populations; it induced genes in the alkaloids and carotenoids biosynthesis pathways in the Mediterranean plants and dihydroflavonols and anthocyanin in the desert plants (Additional file 3: Figure S3).

### Quantitative RT-PCR

**Fig. 7:**
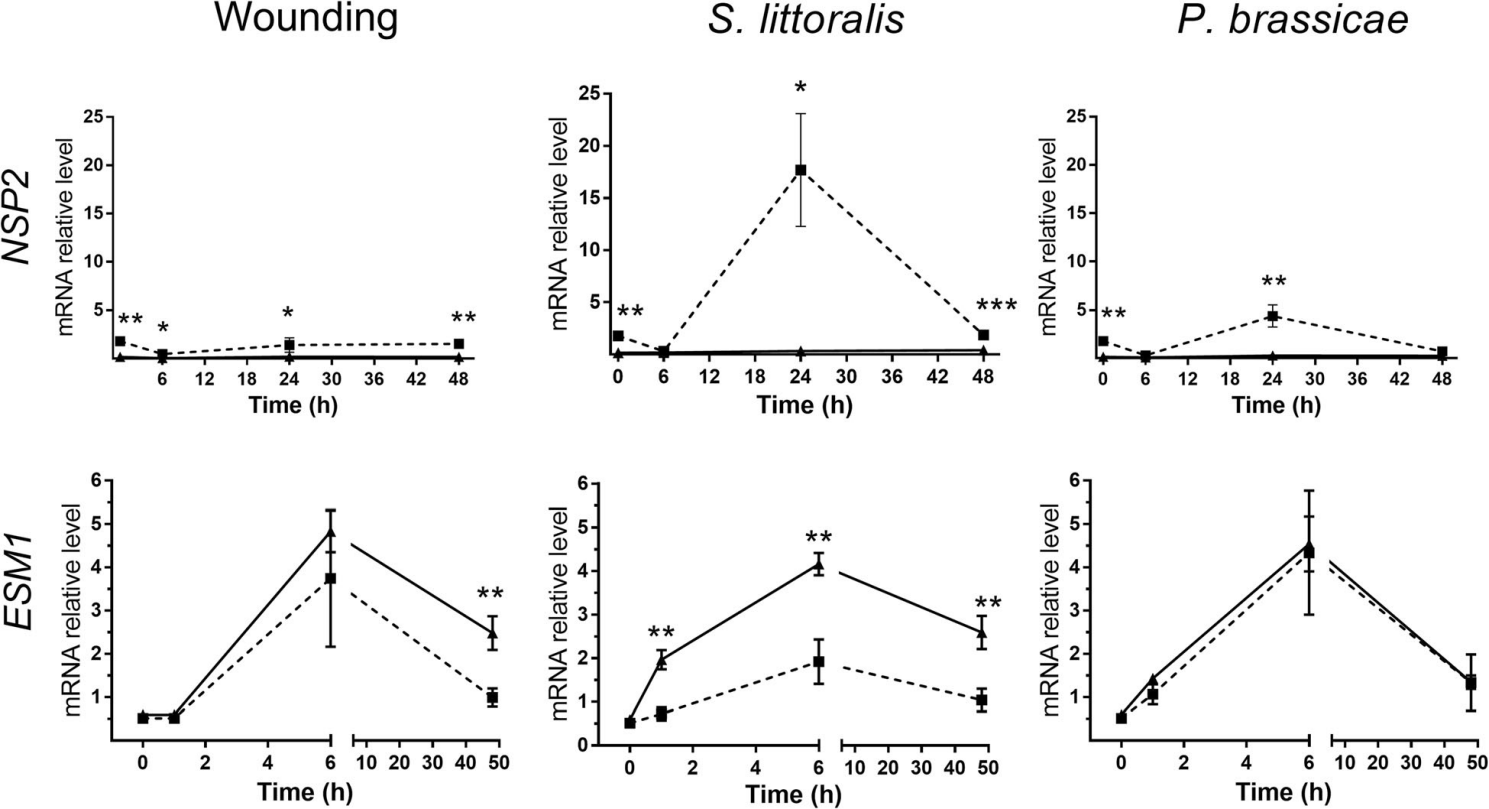
Results of qRT-PCR. Results of qRT-PCR, showing the transcript levels of two candidate genes used to validate the results of the RNA Seq results: the nitrile specifier protein (*NSP2*) and epithio specifier modifier (*ESM1*) involved in glucosinolate breakdown. Results present the average ± SE of five biological replicates in plants of the desert (dashed line) and Mediterranean (solid line) populations following induction of defense by wounding, or application of OS of *S. littoralis* or *P. brassicae* larvae to wounded leaves. Asterisks represent differential expression between plants of the two populations at *P* < 0.05 (^*^) *P* < 0.01 (^**^) and *P* < 0.001 (^***^)

Expression of the genes encoding two myrosinase-associated proteins — *NSP2* and *ESM1* — were measured, because these reflect the division of GS-breakdown products into simple nitriles and ITC, respectively [39, 40]. The uninduced level (control) of *NSP2* was significantly higher in the desert plants than in the Mediterranean ones (*t*-test, *P* < 0.01; Fig. 7). Moreover, significant induction of *NSP2* expression was observed only in desert plants 24 h after treatment with the generalist OS (*t*-test, *P* < 0.05), so that there were significant differences in its expression between plants of the two populations (*t*-test, *P* < 0.05). Significantly higher expression levels in the desert plants than in the Mediterranean ones also were detected 6 and 48 h after wounding (*t*-test, *P* < 0.05 and *P* < 0.01, respectively), and 24 h after application of the specialist herbivore OS (*t*-test, *P* < 0.01; Fig. 7).

Wounding, as well as the application of OS of both herbivores, significantly increased the accumulation of *ESM1* in plants of the two populations 6 h after treatment (*t*-test, *P* < 0.05). Nevertheless, 48 h after wounding, its expression in Mediterranean plants was significantly 2.5 times as high as that in the desert plants (*t*-test, *P <* 0.01; Fig. 7); similarly, significant (*t*-tests, *P* < 0.01) 2.2- and 2.5-fold differences were found 6 and 48 h, respectively, after application of the generalist OS to wounded leaves (Fig. 7). There were no differences between populations in the expression of *ESM1* in response to treatment with the specialist OS.

## Discussion

One of the main findings of this study was that the jasmonic acid and salicylic acid signaling cascades were among the significantly changed pathways that differentiated between the desert and the Mediterranean populations. In the desert plants, OS of both the generalist and specialist herbivores induced the salicylic acid biosynthesis pathway, whereas in the Mediterranean plants herbivory significantly upregulated the jasmonic acid pathway (Figs. 4, 5 and 6). Thus, the differences between the two populations, in their global plant responses to herbivory, such as were demonstrated in the PCA analysis (Fig. 1), with respect to the numbers of down- and upregulated DEGs (Figs. 2 and 3) and to gene clustering (Fig. 5), included changes in the regulatory pathways that could have very important effects on plants’ physiology, growth, and defense responses [41, 42]. The negative crosstalk between the jasmonic and salicylic acid pathways can, therefore, account for the differences between the defense responses of the two populations (Figs. 4, 5, 6 and 7; discussed below).

Defense against insects in members of the Brassicaceae relies mainly on glucosinolates, among which aliphatic glucosinolates were shown to be effective against generalist herbivores [12]. The effects of jasmonic and salicylic acids on the synthesis and accumulation of glucosinolates were shown in *Arabidopsis* e.g. [16, 43, 44]; they typified the antagonistic effects of these phytohormones, especially on gene expression and accumulation of aliphatic glucosinolates [16]. Similarly, the aliphatic glucosinolates synthesis pathway was upregulated in plants of the Mediterranean population (Fig. 4a), whereas it was significantly downregulated by the three elicitation treatments in plants of the desert population (Fig. 4b). Furthermore, the RNA-Seq results (Fig. 4) and qRT-PCR analysis (Fig. 7) indicated that differences in responses to herbivory between populations of *E. sativa* included differences in the expression of glucosinolate breakdown genes: OS of the generalist herbivore caused higher expression of *ESM1* in Mediterranean plants than in desert ones (Fig. 7). Since *ESM* was shown to repress nitrile formation in *Arabidopsis* in favor of release of more harmful ITC [45], the present results support our previous opinion [5] that accumulation of aliphatic glucosinolates and ITC breakdown products plays the primary direct defensive role against herbivores in plants of the Mediterranean population. The activation of a combination of defensive traits was evident in plants of the Mediterranean population, when all three elicitation treatments induced the mevalonate pathway (Figs. 4 and 6), which might indicate an additional indirect defense [46].

In general, responses of plants of the Mediterranean population to *S. littoralis* were substantially stronger than those of the desert plants (Figs. 3 and 5B). Defense responses of the desert plants included upregulation of synthesis of flavonoids, known for their role in direct defense against herbivores [47], and of mechanical defenses (suberin and lignin synthesis pathways) (Figs. 4 and 6) that strengthen the physical barrier of the cell wall. Moreover, the induction of putrescine biosynthesis in response to OS of the generalist insect (Fig. 4a) possibly indicated involvement of polyamines in wound healing [48], but also might suggest that the response in plants of the desert population involves sclerophylly. Thus, the role of flavonols and mechanical barriers in induced defenses in the desert plants (Fig. 5b) may be associated with their dual role in defense against biotic and abiotic stresses [49].

All three elicitation treatments induced higher expression of *NSP2* in plants of the desert population than in Mediterranean plants (Fig. 7). It previously was shown that the release of nitriles provided crucifer plants with the ability to avoid herbivory by specialist herbivores, which employ ITC as recognition cues to detect their host plants [50, 51]. Our observations indicate that the specialist *Plutella xylostela* was more abundant in the desert monospecific habitat than in the heterogeneous Mediterranean plant community [52]. Accordingly, it can be deduced that the direction of glucosinolate breakdown to producing simple nitriles is efficient against *P. xylostela*, whereas glucosinolates and their breakdown products ITC provide efficient defense against generalist herbivores in the Mediterranean site.

According to the generalist vs. specialist herbivore paradigm, the ability to perceive herbivory enables plants to respond differently to different herbivores. As a result, in the evolutionary battle against specialist herbivores, various plant defense mechanisms are induced to act against adapted insects [53]. It previously was shown that accumulation of flavonols in *A. thaliana* increased plant resistance to larvae of the specialist *P. brassicae* [54]. In the present study all the results indicate that both populations responded differently to the three defense-elicitation treatments (Figs. 1, 2, 3, 4, 5, 6 and 7; Additional file 3: Figure S3). Furthermore, elicitation with OS of *P. brassicae* resulted in the induced synthesis of simple coumarins in plants of both populations (clade D1; Figs. 5 and 6); activation of alkaloid and carotenoid synthesis in Mediterranean plants, and synthesis of dihydroflavones and anthocyanin in desert plants (Figs. 5 and 6; Additional file 3: Figure S3). That these pathways were not induced by OS of *S. littoralis* or by wounding (Additional file 3: Fig. S3) indicates the ability of plants of both populations to perceive different herbivores and to maximize their defenses and fitness in accordance with the type of attack. It is interesting to note that OS of the specialist herbivore induced genes of the aromatic glucosinolate pathway in plants of both populations (Fig. 6c). Since the metabolism of aromatic glucosinolate to benzyl cyanide increases the vulnerability of eggs to parasitic wasps [55], our results highlight the role of indirect defenses after an attack by specialist herbivores.

## Conclusion

In summary, the result of the transcriptome screening indicated ecotypic differentiation between two defense strategies in populations of *E. sativa* originating from desert and Mediterranean habitats, respectively. The fact that the defense response is governed by differences between regulatory signaling pathways, i.e., jasmonic and salicylic acid pathways, might indicate global *in-planta* differentiation that can be associated with environmental adaptation. In *Boechera* sp., interspecific variation in induced defenses against herbivores was associated with differences in their adaptation to drought [56]. Accordingly, the role of flavonols and mechanical defenses in induced defense in plants of the desert population may be associated with their dual role in defense against biotic and abiotic stresses [49]. In addition, we suggest that differentiation between the defense responses in the two populations is directly linked to biotic selection agents [52]. Thus, the direction of glucosinolate breakdown to production of simple nitriles, and activation of multiple alternative pathways provide efficient defense against both specialist and generalist insects in the desert habitat.

## Supplementary information

**Additional file 1: Figure S1.** Comparison of the resulting 80,946 contigs against the transcriptome catalogue.

**Additional file 2: Figure S2.** Results of the principal component analysis (PCA) of the transcriptome profiles of the three replicates of the desert and Mediterranean (Med) populations. Control, early (ER) and late (LR) responses to the three elicitation treatments: wounding (Wo) or OS of *S. littoralis* (*Sl*) or *P. brassicae* (*Pb*).

**Additional file 3: Figure S3.** MapMan illustrations of the upregulated defense pathways in plants of the Mediterranean (blue) and the desert (red) populations in response to elicitation by wounding (A), or OS of *S. littoralis* (B) and *P. brassicae* (C). The upper and lower rows represent the early and late responses, respectively.

**Additional file 4.** Statistical summary of the transcriptome catalogue.

**Additional file 5. **List of exclusive up- and downregulated pathways of the desert and Mediterranean plants. The list present the pathway and strength of *P* values of leaves that were elicited by wounding and by OS treatments.

**Additional file 6.** Categorization to pathways of transcripts designated to different clades.

## Data Availability

All relevant data supporting our findings is provided in the article and supporting information. The sequencing data were deposited in the NCBI as detailed in the Methods section.

## Abbreviations

DEG: Differentially regulated genes
ER: Early response
ESM: Epithio specifier modifier
GO: Gene ontology
ITC: Isothiocyanates
LR: Late response
NSP: Nitrile-specifier protein
OS: Oral secretions
PCA: Principal component analysis
qRT-PCR: quantitative real-time PCR
