# Supplementary figures and images for: Transcriptome responses to different herbivores reveal differences in defense strategies between populations of Eruca sativa
Source: BMC Genomics. 2019 Nov 12;20:843. doi: 10.1186/s12864-019-6217-9 (PMC6852892; doi:10.1186/s12864-019-6217-9)

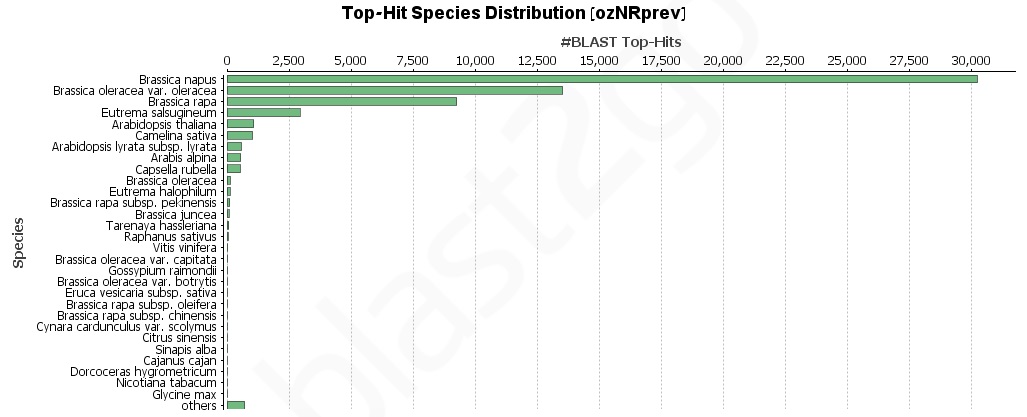

Supplement: Supplementary file 4 — Additional file 4. Statistical summary of the transcriptome catalogue. [file 12864_2019_6217_MOESM4_ESM.jpg]

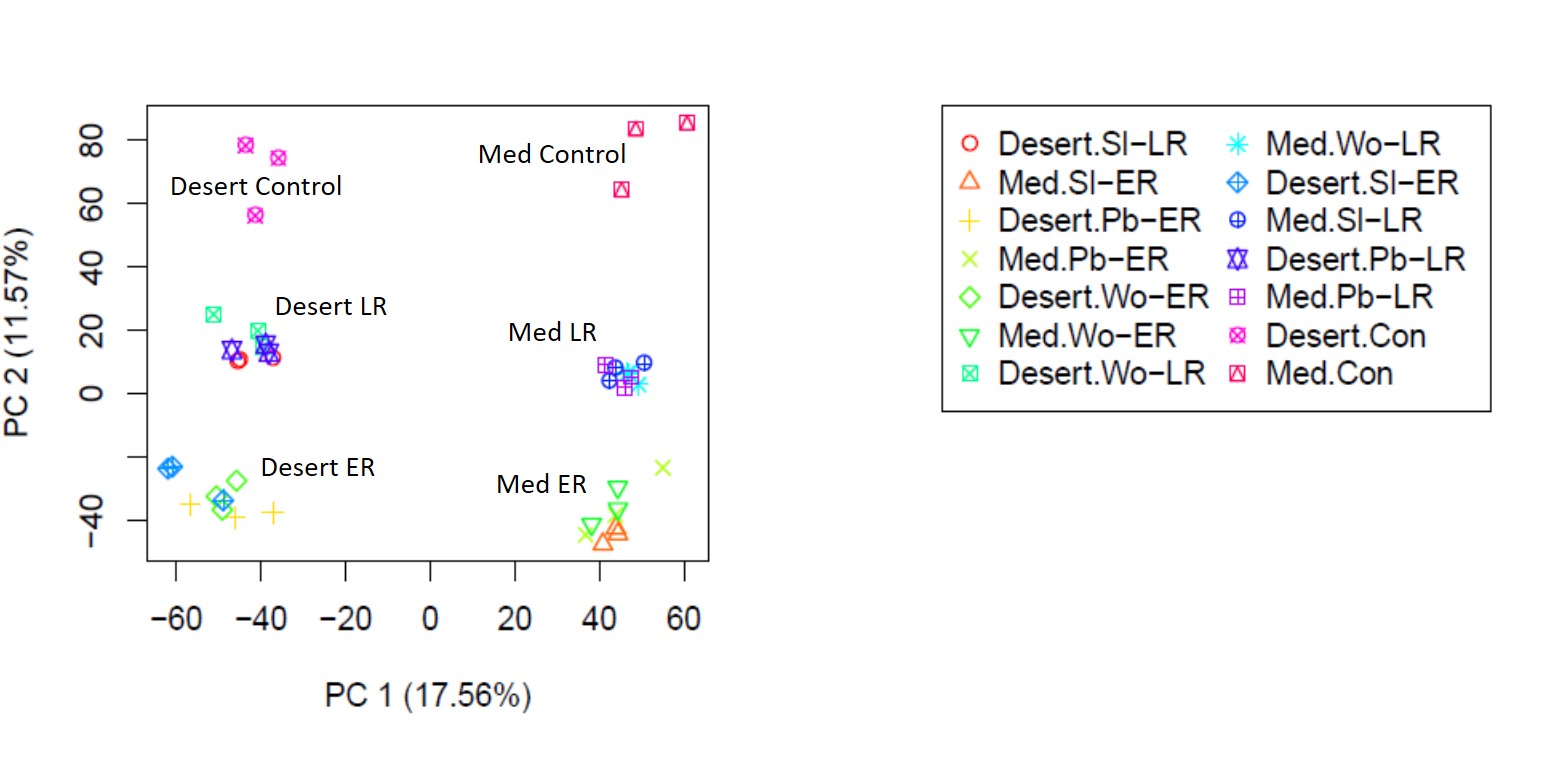

Supplement: Supplementary file 5 — Additional file 5. List of exclusive up- and downregulated pathways of the desert and Mediterranean plants. The list present the pathway and strength of P values of leaves that were elicited by wounding and by OS treatments. [file 12864_2019_6217_MOESM5_ESM.jpg]

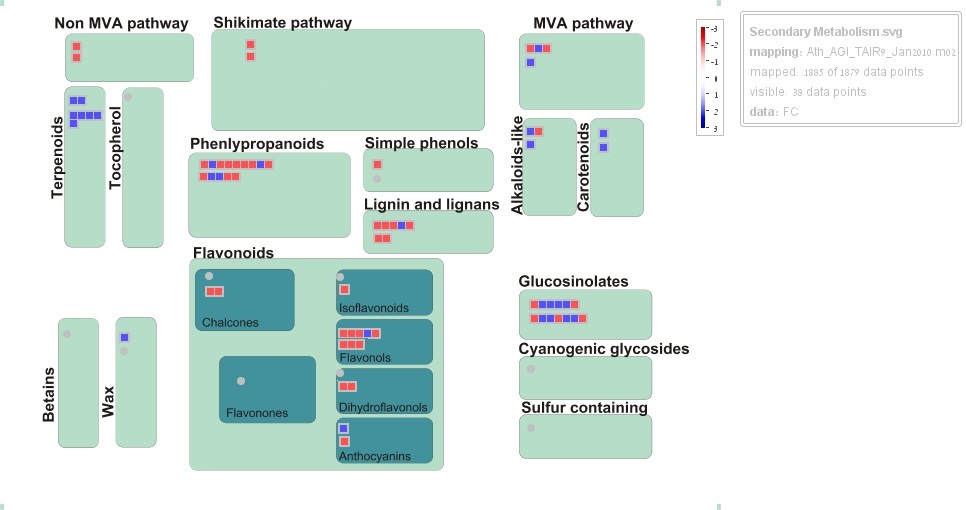

Supplement: Supplementary file 6 — Additional file 6. Categorization to pathways of transcripts designated to different clades. [file 12864_2019_6217_MOESM6_ESM.zip › Figure S3C.jpg]

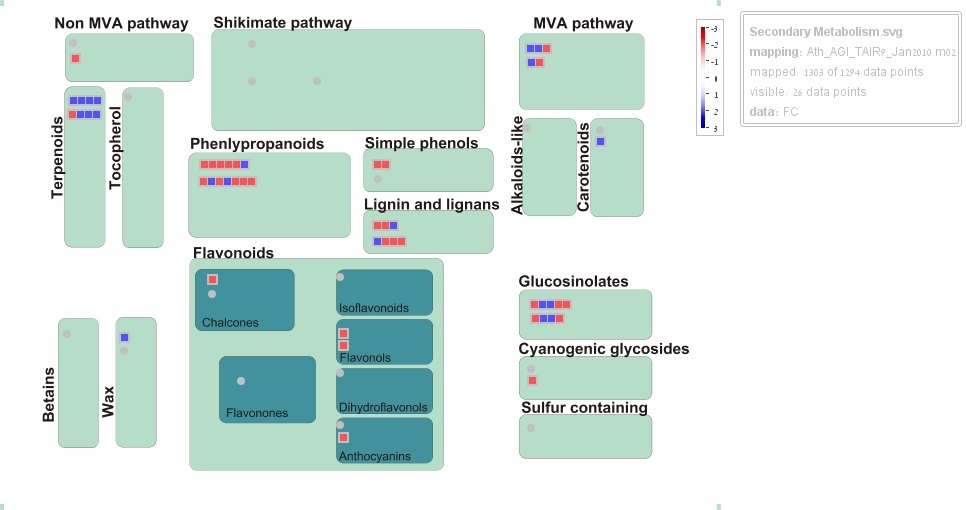

Supplement: Supplementary file 6 — Additional file 6. Categorization to pathways of transcripts designated to different clades. [file 12864_2019_6217_MOESM6_ESM.zip › Figure S3B.jpg]

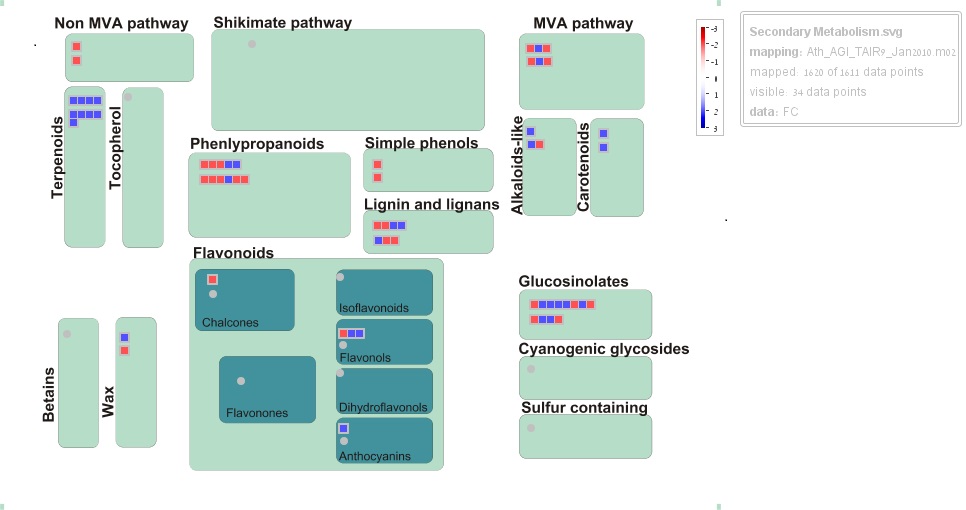

Supplement: Supplementary file 6 — Additional file 6. Categorization to pathways of transcripts designated to different clades. [file 12864_2019_6217_MOESM6_ESM.zip › Figure S3A.jpg]
